# Effective social spider optimization algorithms for distributed assembly permutation flowshop scheduling problem in automobile manufacturing supply chain

**DOI:** 10.1038/s41598-024-57044-8

**Published:** 2024-03-16

**Authors:** Weiwei Zhang, Jianhua Hao, Fangai Liu

**Affiliations:** 1 School of Science, Shandong Jiaotong University, Jinan, 250357 China; 2 School of Information Science and Engineering, Shandong Normal University, Jinan, 250014 China

**Keywords:** Mathematics and computing, Computer science

## Abstract

This paper presents a novel distributed assembly permutation flowshop scheduling problem (DAPFSP) based on practical problems in automobile production. Different from the existing research on DAPFSP, this study considers that each component of the final product is composed of more than one part. Components are processed in a set of identical components manufacturing factories and are assembled into products in the assembly factory. The integration of manufacturing processes is an important objective of Industry 4.0. For solving this problem with the minimum makespan criterion, we introduce a three-level representation and a novel initialization method. To enhance the search ability of the proposed algorithms, we design three local search methods and two restart procedures according to characteristics of the problem. Then, by incorporating the problem specific knowledge with the social spider optimization algorithm (SSO), we propose three SSO variants: the SSO with hybrid local search strategies (HSSO), the HSSO with restart procedures (HSSOR), and the HSSOR with self-adaptive selection probability (HSSORP). Finally, 810 extended instances based on the famous instances are used to test the proposed algorithms. In most cases, HSSOR performs the best, with an average comparison metric value of 0.158% across three termination conditions, while the average comparison metric value for the best comparison method is 2.446%, which is 15.481 times that of HSSOR. Numerical results demonstrate that the proposed algorithms can solve the problem efficiently.

## Introduction

Assembly production systems are widely used in various industries, e.g., in electronic, construction machinery, automobile and auto parts industries. In order to boost the production efficiency of assembly systems, researchers and industrial engineers have paid great attention to it in the past decades. The assembly flowshop scheduling problem (AFSP) was firstly presented by Lee et al.^1^ Later, Potts et al.^2^ illustrated the two-stage assembly flowshop problem (TSAFSP) according to the production of personal computers and proved that the general version of the considered problem is NP-hard. Koulamas and Kyparisis^3^ introduced the three-stage assembly flowshop scheduling problem taking the immediate transportation operation into consideration. Due to the practicality and complexity of AFSP, many literatures have conducted deep researches to it^4–10^.

Recently, with the trend of globalization in the manufacture, considerable attention is paid to distributed flowshop scheduling problems since Naderi’s innovative paper^11^. Later, Hatami et al.^12,13^ first introduced the distributed assembly permutation flowshop scheduling problem (DAPFSP) and proposed effective algorithms to study the modern supply chain. DAPFSP is composed by two stages: component processing and assembly. In the first stage, components are processed in several factories with the identical equipment. The latter stage is to assemble components processed in the first stage into products. As the widespread use of DAPFSP in modern supply chains and the NP-hardness of large-scale DAPFSP, many researchers investigated it in depth and proposed heuristics to achieve near-optimum solutions within reasonable time. Harami, Ruiz and Andres-Romano^14^ studied DAPFSP with sequence dependent setup times and developed two constructive heuristics to obtain efficient initial solutions. Li et al.^15^ employed the genetic algorithm (GA) for the DAPFSP, which combining an improved crossover strategy and three novel local search strategies. Wang, Zhang and Liu^16^ incorporated the variable neighborhood search (VNS) into the memetic algorithm (MA) for the DAPFSP with the makespan criterion. Moreover, Deng et al.^17^ propose a competitive memetic algorithm for the distributed TSAFSP (DTSAFSP). Lin and Zhang^18^ hybridized the biogeography-based optimization (BBO) algorithm with several heuristics to minimize the makespan of the DAPFSP. A novel estimation of distribution algorithm based memetic algorithm (EDAMA) was developed by Wang and Wang^19^ to minimize the makespan of DAPFSP. Lin, Wang and Li^20^ proposed the backtracking search hyper-heuristic (BS-HH) to figure out the DAPFSP. The effectiveness of BBO, EDAMA and BS-HH was demonstrated on the benchmark instances generated by Hatami et al.^12^. In addition, Gonzalez-Neira et al.^21^ considered the DAPFSP with stochastic processing times. More recently, several papers carried further research on the DAPFSP. Ruiz, Pan and Naderi^22^ proposed iterated greedy (IG) methods for the DAPFSP with makespan criterion. Ferone et al.^23^ developed a biased-randomized iterated local search for the same problem. Pan et al.^24^ presented an extension of the DAPFSP and proposed effective heuristics to solve the considered scheduling problem. Recently, Sang et al.^25^ studied the DAPFSP proposed by Pan et al.^24^ with the total flowtime criterion and proposed three diffident discrete invasive weed optimization (DIWO).

Studies in DAPFSP suppose that each component of the final product consists of only one part but one component could be more complicated and composed of several parts in the practical production. Meanwhile, with the trend of production specialization and internationalization, one factory generally does not engage in all stages of production from raw materials to final products, and the production of semi-finished products is usually completed by other factories. In many practical production scenarios, especially in the automobile production process, one semi-finished product is usually a large-sized component composed of many small parts. For example, in automotive enterprises such as Anhui Jianghuai Automobile Co., Ltd and Jiangling Motors Corporate of China, components from other manufacturers are added to the truck frame on the final assembly line, such as the engine^24^, the axle, the transmission shaft, and so on. Each of these large-sized components is composed of some small parts and can be considered as semi-finished products produced by other components manufacturing factories. To the best of our knowledge, there is no study on DAPFSP considering that each component of the final product is composed of several parts, but this problem exists in practical production. For this problem, efficient scheduling can improve the production efficiency of enterprises, reduce resource waste and pollutant emissions, and help enterprises achieve green production. Therefore, this paper considers the practical situation in the automobile manufacturing supply chain and addresses a novel DAPFSP based on the innovative work of Hatami et al.^12,13^ and Pan et al.^24^. There are several optimization objectives in the flowshop scheduling problem^26,27^, such as total flow time, total waiting time, makespan, and weighted flow time. This paper aims to minimize makespan of the proposed problem due to the time of delivery is an important content in trade contracts. It has been proved that minimizing makespan of DAPFSP is NP-hard, hence, the presented problem is NP-hard because of more complexity.

Over recent years, various meta-heuristics have been proposed and applied to solve production scheduling problems, such as genetic algorithm (GA)^28^, particle swarm optimization (PSO)^29–31^, artificial bee colony (ABC)^32–34^, firefly algorithm (FA)^35,36^, grey wolf optimizer (GWO)^37,38^, whale optimization algorithm (WOA)^39–42^, migrating birds optimization algorithm (MBO)^43^, and so on. Recently, the social spider optimization (SSO), inspired by the cooperative behavior of spiders, is an emerging and excellent swarm intelligent algorithm. The SSO was first proposed by Cuevas and Cienfuegos^44^ for solving constrained optimization problems. To enhance the search ability of SSO, Klein et al.^45^ introduced a modified SSO and applied it to electromagnetic optimization. Nguyen^46^ presented a novel improved SSO (NISSO) for optimal power flow problem. Zhang and Xing^47^ developed a memetic SSO (MSSO) for the distributed TSAFSP. Inspired by many successful applications of SSO, we adopt SSO to solve the DAPFSP proposed in this paper.

Considering the complexity of the proposed DAPFSP in this paper, we introduce a three-level representation and present three shift operators for it. According to the three shift operators, three local search methods are designed respectively. Two restart procedures are presented to maintain the diversity of the population and avoid premature convergence. We combine the problem specific knowledge with the SSO and propose three SSO variants. Experimental results based on 810 extended instances demonstrate the effectiveness of our algorithms.

The remainder of our paper is organized as follows. “Problem definition and formulation” section illustrates the proposed DAPFSP in this paper and formulates the proposed problem with makespan criterion. “The canonical SSO algorithm” section shows the concept and theory of SSO. “The proposed three social spider optimization algorithms” section reports three variants of SSO in detail. In “Experiment analyses” section, we conduct experiments and analyze the results. Finally, “Conclusions” section details conclusions and our future research work.

## Problem definition and formulation

### Problem definition

**Figure 1:**
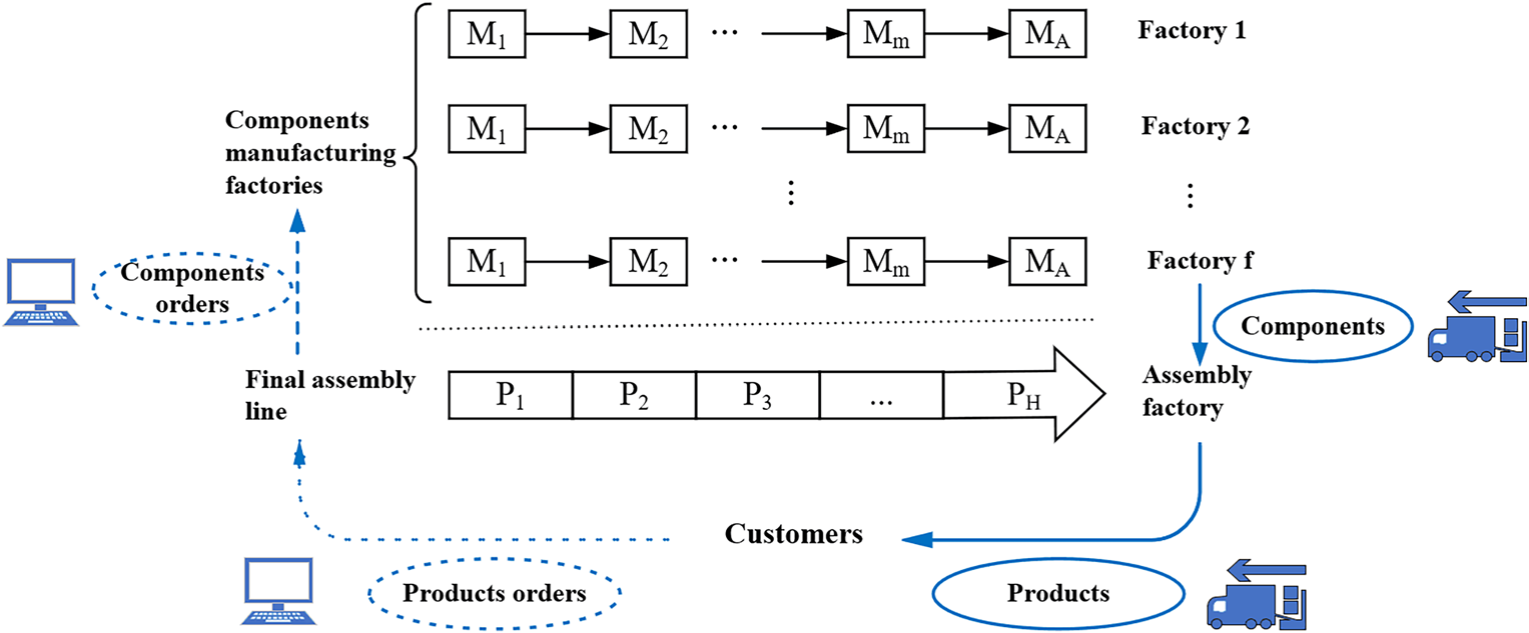
Illustration of the presented DAPFSP.

This section briefly illustrates the presented DAPFSP similar to examples reported by Hatami et al.^12^ and Pan et al.^24^. There are one assembly factory and a set of $F$ identical components manufacturing factories (See in Fig. 1). Under the Make-To-Order (MTO) mode of automobile production, the customer places an order for *H* products to the assembly factory, and then the assembly factory orders components from *F* components manufacturing factories according to the customer’s order. Each final product is composed of several components and each component can be manufactured in any of the $F$ components manufacturing factories. Each component manufacturing factory $f$ ($f = 1, \ldots ,F$) is formed by the same production stage and an assembly machine. The production stage is a flowshop and consists of $m$ machines. In the flowshop, every part must be processed by $m$ machines in sequence, i.e., $M_{1}$, $M_{2}$, and so on until $M_{m}$. All parts belonging to one component must be processed continuously in one factory. In the assembly stage of one component manufacturing factory, all parts belonging to the same component are assembled on the assembly machine $M_{A}$. Finished components are transported to the final assembly factory. Finally, *H* products $\left\{ {P_{1} ,P_{2} , \ldots ,P_{H} } \right\}$ are assembled on the final assembly line in the assembly factory and delivered to customers within the delivery date.

### Problem formulation

Following assumptions are used to formulate the considered DAPFSP:

- All parts of each component are available to be processed at time zero.
- One component must be processed in one components manufacturing factory. Changing factory is not allowed.
- The transportation time within and between factories is ignored.
- The assembly operation of one component can be performed if all parts belong to the component are processed.
- When all components of a product have been processed, the assembly operation for that product can begin.

Parameters, indices, sets, and variables are listed as follows.

Parameters:

**Table:** 

*F*	Number of components manufacturing factories
*M*	Number of machines in the production stage
*α*	Number of products
*β*	Number of all the components
*Χ*	Number of all the parts
*n*_*p*_	Number of the components belonging to product $\Delta_{p}$,$p = 0,1,2, \ldots ,\alpha \cdot n_{0} = 0$
*u*_*c*_	Number of the parts belonging to component $\omega_{c}$, $c = 0,1,2, \ldots ,\beta \cdot u_{0} = 0$.$Bj,m$
$P_{j,m}$	Processing time of part *j* on machine *m*
$A_{{\omega_{c} }}$	Assembly time of component $\omega_{c}$
$A_{{\Delta_{p} }}$	Assembly time of product $\Delta_{p}$
*T*	A very large positive number

Indices:

**Table:** 

*f*	Actory index
*i j*	Part index
*c c*′	Component index
*p p*′	Product index
*m*	Index of machines in the production stage

Sets:

**Table:** 

$\Delta = \left\{ {\Delta_{1} ,\Delta_{2} , \ldots ,\Delta_{\alpha } } \right\}$	Set of products
$\Delta_{0}$	A dummy product
$\Lambda_{p} = \left\{ {\Lambda_{p,1} ,\Lambda_{p,2} , \ldots ,\Lambda_{{p,n_{p} }} } \right\}$	Set of components belonging to $\Lambda_{p}$
$\Lambda = \left\{ {\omega_{{k_{0} + 1}} ,\omega_{{k_{0} + 2}} , \ldots \omega_{{k_{1} }} ,\;\omega_{k + 1} , \ldots ,\omega_{{k_{\alpha - 1} + 1}} , \ldots \omega_{{k_{\alpha } }} } \right\}$	Set of all the components, where components $\omega_{{k_{p - 1} + 1}} , \ldots \omega_{{k_{p} }}$ belonging to the product $\Delta_{p} \cdot k_{0} = 0,\;k_{p} = \sum\nolimits_{s = 1}^{p} {n_{s} }$
$\omega_{0}$	A dummy component
$\theta_{0}$	A dummy part
$\delta_{c} = \left\{ {\delta_{c,1} ,\delta_{c,2} , \ldots \delta_{{c,u_{c} }} } \right\}$	Set of parts belonging to *ω*_*c*_
$\delta = \left\{ {\theta_{{z_{0} + 1}} ,\theta_{{z_{0} + 2}} , \ldots \theta_{{z_{1} }} ,\;\theta_{{z_{1} + 1}} , \ldots \theta_{{z_{\beta - 1} + 1}} , \ldots \theta_{{z_{\beta } }} } \right\}$	Set of all the parts, where parts $\theta_{{z_{c - 1} + 1}} , \ldots \theta_{{z_{c} }} ,$ belonging to the component $\omega_{c} \cdot z_{0} = 0,\;z_{c} = \sum\nolimits_{s = 1}^{c} {u_{s} }$

Variables:

**Table:** 

$B_{j,m}$	Beginning time of part $j$ on machine $m$
$C_{j,m}$	Completion time of part $j$ on machine $m$
$C_{{\omega_{c} }}$	Completion time of component $\omega_{c}$
$C_{{\Delta_{p} }}$	Completion time of product $\Delta_{p}$
$C_{\max }$	Makespan of all products

Binary variables:

$$X_{i,j} = \left\{ {\begin{array}{*{20}l} 1 \hfill & {{\text{if}}\;{\text{part}}\;j\;{\text{is}}\;{\text{processed}}\;{\text{immediately}}\;{\text{after}}\;{\text{part}}\;i} \hfill \\ 0 \hfill & {{\text{otherwise}}} \hfill \\ \end{array} } \right.$$

$$Y_{c,f} = \left\{ {\begin{array}{*{20}l} 1 \hfill & {{\text{if}}\;{\text{component}}\;c\;{\text{is}}\;{\text{process}}\;{\text{ed}}\;{\text{in}}\;{\text{factory}}\;f} \hfill \\ 0 \hfill & {{\text{otherwise}}} \hfill \\ \end{array} } \right.$$

$$Z_{{c,c^{\prime } }} = \left\{ {\begin{array}{*{20}l} 1 \hfill & {{\text{if}}\;{\text{component}}\;c\;{\text{is}}\;{\text{assembled}}\;{\text{immediately}}\;{\text{after}}\;{\text{component}}\;c^{\prime } \;{\text{on}}\;{\text{the}}\;{\text{assembly}}\;{\text{machine}}} \hfill \\ 0 \hfill & {{\text{otherwise}}} \hfill \\ \end{array} } \right.$$

$$H_{{p,p^{\prime } }} = \left\{ {\begin{array}{*{20}l} 1 \hfill & {{\text{if}}\;{\text{product}}\;p\;{\text{is}}\;{\text{assembled}}\;{\text{immediately}}\;{\text{after}}\;{\text{product}}\;p^{\prime } \;{\text{on}}\;{\text{the}}\;{\text{final}}\;{\text{assembly}}\;{\text{factory}}} \hfill \\ 0 \hfill & {{\text{otherwise}}} \hfill \\ \end{array} } \right.$$

The mathematical model of the presented DAPFSP can be formulated as follows:

1 $$\min \;C_{\max }$$

2 $$\begin{aligned}&\text{S. T}\\ &\sum\limits_{i = 0,i \ne j}^{\chi } {X_{i,j} } = 1,\quad j = 1,2, \ldots ,\chi \end{aligned}$$

3 $$\sum\limits_{j = 0,j \ne i}^{\chi } {X_{i,j} } = 1,\quad i = 1,2, \ldots ,\chi$$

4 $$\sum\limits_{f = 1}^{F} {Y_{c,f} } = 1,\quad c = 1,2, \ldots ,\beta$$

5 $$\sum\limits_{i = 1}^{{z_{l - 1} }} {\sum\limits_{{j = z_{l - 1} + 1}}^{{z_{l} }} {X_{i,j} } } + \sum\limits_{{i = z_{l} + 1}}^{{z_{\beta } }} {\sum\limits_{{j = z_{l - 1} + 1}}^{{z_{l} }} {X_{i,j} } } = 1,\quad l = 1,2, \ldots ,\beta$$

6 $$C_{j,m} \ge C_{j,m - 1} + P_{j,m} ,\quad j = 1,2, \ldots ,\chi ,\;m = 2,3, \ldots ,M$$

7 $$C_{j,m} \ge C_{i,m} + P_{i,m} + (X_{i,j} - 1) \cdot T,\quad i = 1,2, \ldots ,\chi ,\;j = 1,2, \ldots ,\chi ,\;m = 1,2, \ldots ,M$$

8 $$C_{{\omega_{c} }} \ge C_{j,M} + A_{{\omega_{c} }} ,\quad c = 1,2, \ldots ,\beta ,\;j = z_{l - 1} + 1, \ldots ,z_{l}$$

9 $$C_{{\omega_{c} }} \ge C_{{\omega_{{c^{\prime } }} }} + A_{{\omega_{c} }} + (Z_{{c,c^{\prime } }} - 1) \cdot T,\quad c = 1,2, \ldots ,\beta ,\;c^{\prime } = 1,2, \ldots ,\beta ,\;c \ne c^{\prime }$$

10 $$C_{{\Delta_{p} }} \ge C_{{\omega_{c} }} + A_{{\Delta_{p} }} ,\quad p = 1,2, \ldots ,\alpha ,\;c = k_{p - 1} + 1, \ldots ,k_{p}$$

11 $$C_{{\Delta_{p} }} \ge C_{{\Delta_{{p^{\prime } }} }} + A_{{\Delta_{p} }} + (H_{{p,p^{\prime } }} - 1) \cdot T,\;p = 1,2, \ldots ,\alpha ,\;p^{\prime } = 1,2, \ldots ,\alpha ,\;p \ne p^{\prime }$$

12 $$C_{\max } \ge C_{{\Delta_{p} }} ,\quad p = 1,2, \ldots ,\alpha$$

13 $$Xi,j \in \left\{ {0,1} \right\},\quad \forall i,j,i \ne j$$

14 $$Y_{c,f} \in \left\{ {0,1} \right\},\quad \forall c,f$$

15 $$Z_{{c,c^{\prime } }} \in \left\{ {0,1} \right\}\quad \forall c,c^{\prime } ,c \ne c^{\prime }$$

16 $$H_{{p,p^{\prime } }} \in \left\{ {0,1} \right\},\;\forall p,p^{\prime } ,p \ne p^{\prime } .$$

Formulation (1) illustrates the objective is minimizing the makespan of all products. Constraint sets (2) and (3) mandate that every part has only one immediate successor and one immediate predecessor. Constraint set (4) ensures that each component must be assigned to only one factory. Constraint set (5) enforces that the parts belonging to the same component should be processed in succession and cannot be separated. Constraint set (6) makes sure that a part cannot start until the previous operation is completed. Constraint set (7) indicates that one machine can only process a part at a time. Constraint set (8) guarantees that the assembly operation of a component can only be started after all parts belonging to it are completed. Constraint set (9) enforces that the assembly machine cannot assemble two components at the same time. Constraint set (10) requires that one product cannot be assembled in the final assembly factory before all the components of the product are completed. Constraint set (11) indicates that the final assembly factory cannot assemble two products at a time. Constraint set (12) defines the makespan of all products. Constraint sets (13)-(16) define the domain of decision variables.

### An illustrative example

**Figure 2:**
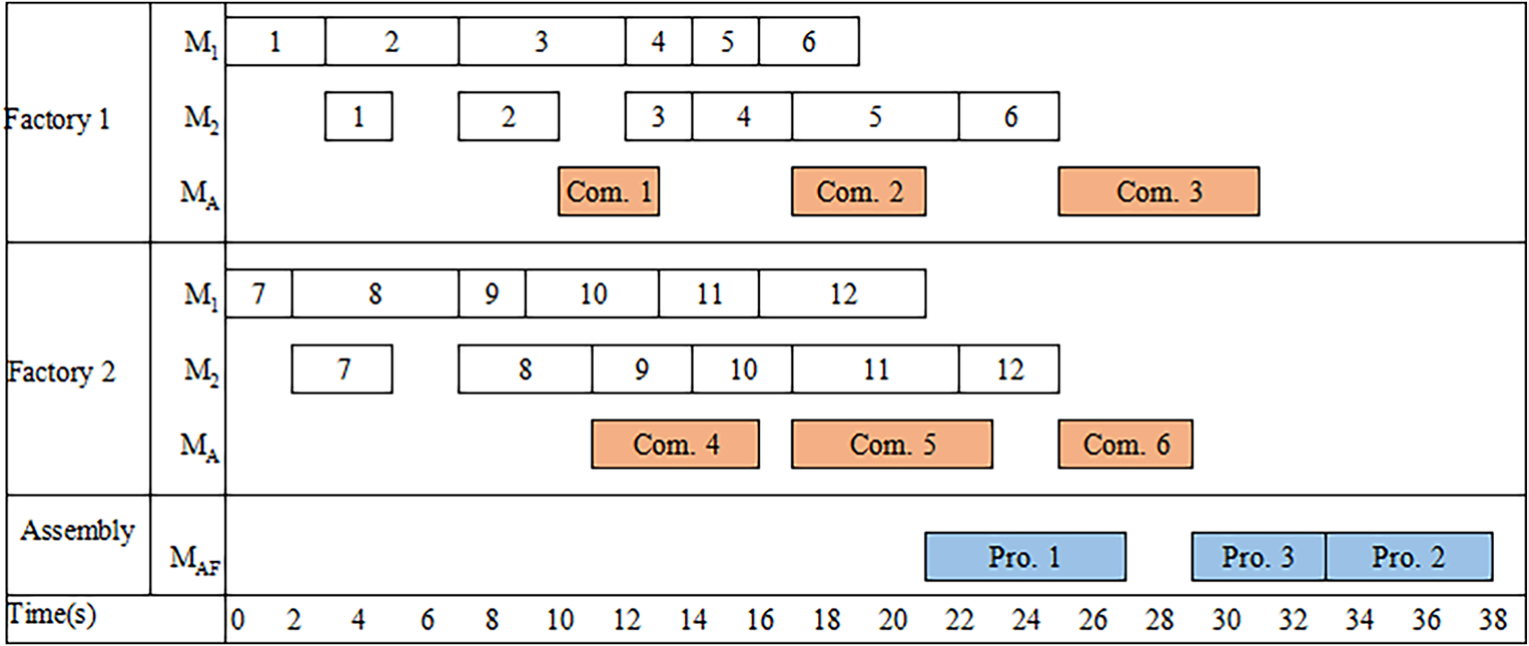
Gantt chart of the example.

Sequence-based variables are used with a set of *min {F, α, β}* + 1 dummy parts. Consider an example with $F = 2$, $M = 2$, $\alpha = 3$, $\beta = 6$, $\chi = 12$, $\Lambda_{1} = \{ 1,2\}$, $\Lambda_{2} = \{ 3,4\}$, $\Lambda_{3} = \{ 5,6\}$, $\delta_{1} = \{ 1,2\}$, $\delta_{2} = \{ 3,4\}$, $\delta_{3} = \{ 5,6\}$, $\delta_{4} = \{ 7,8\}$, $\delta_{5} = \{ 9,10\}$, $\delta_{6} = \{ 11,12\}$. The processing time of products, components and parts are shown in Table 1. One feasible solution is *X*_0,1_ = *X*_1,2_ = *X*_2,3_ = *X*_3,4_ = *X*_4,5_ = *X*_5,6_ = *X*_6,0_ = *X*_0,7_ = *X*_7,8_ = *X*_8,9_ = *X*_9,10_ = *X*_10,11_ = *X*_11,12_ = *X*_12,0_ = 1, where 0 is the dummy part. The part sequence is {0, 1, 2, 3, 4, 5, 6, 0, 7, 8, 9, 10, 11,12, 0}, where the part sequence {1, 2, 3, 4, 5, 6} and {7, 8, 9, 10, 11,12} are assigned to the factory 1 and 2, respectively. Then the component processing sequence is {1, 2, 3} and {4, 5, 6} in the factory 1 and 2, respectively. Finally, the finished components are transported to the assembly factory to be assembled into products. In the assembly factory, product sequence is {1, 3, 2} and the makespan of the example is 38 (see in Fig. 2).

**Table 1:** Processing time for all parts, components and products.

Part	1	2	3	4	5	6	7	8	9	10	11	12
*M*_1_	3	4	5	2	2	3	2	5	2	4	3	5
*M*_2_	2	3	2	3	5	3	3	4	3	3	5	3
Component	1		2		3		4		5		6	
*M*_*A*_	3		4		6		5		6		4	
Product	1				2				3			
*M*_*AF*_	6				5				4

## The canonical SSO algorithm

The concept and theory of the social spider optimization algorithm was developed by Cuevas and Cienfuegos^44^ which mathematically models the behaviour of social spiders. The validity of SSO has been proved in many different optimization problems^45–47^.

The search space of SSO is regarded as a communal web. Social spiders use the communal web as a communication media to perform cooperative behavior^48^, such as mating, prey capturing and social contact^49^. Different from most of existing swarm algorithms, the SSO divides the social spider population into two different groups: the female group and the male group. According to gender, each social spider is executed by a different set of evolution operators. Moreover, SSO adopts mating operator to exchange information among two groups and produce offspring. These operators enhance the search ability of SSO to find the optimal solution. In this way, the SSO can avoid premature convergence and keep the balance between exploration and exploitation.

## The proposed three social spider optimization algorithms

The canonical SSO cannot be used directly for discrete optimization problem because it is designed to solve continuous optimization problem. Therefore, this section presents three discrete SSO algorithms for the proposed DAPFSP in this paper. We first introduce the solution representation, the initialization method, cooperative operators, three neighborhood operators, three local search methods, two restart producers, and present three discrete SSO algorithms in detail.

### Solution representation

Solution representation is an important part in the design of scheduling algorithms. We design a three-level representation for the DAPFSP presented in this paper. The representation includes three parts: a product sequence, $\alpha$ component sequences and $\beta$ part sequences. The product sequence $\pi$ introduce the processing sequence of all the products in the assembly factory. In components manufacturing factories, the components belonging to products are assigned according to the product sequence. In other words, the components of the first product in the sequence are assigned first. And then, the components belonging to the second product in the sequence are considered. The part sequence of a component is a permutation of all the parts belonging to the component. Suppose a solution *s*, its product sequence can be expressed as $\pi = \left\{ {\pi_{1} ,\pi_{2} , \ldots ,\pi_{\alpha } } \right\}$ ($\pi_{1}$: the first product of the product sequence). The component sequence of product $\pi_{k}$ is $\Lambda_{{\pi_{k} }} = \{ \Lambda_{{\pi_{k} ,1}} ,\Lambda_{{\pi_{k} ,2}} , \ldots ,\Lambda_{{\pi_{k} ,n_{{\pi_{k} }} }} \}$, where $n_{{\pi_{k} }}$ is the number of components belonging to product $\pi_{k}$. The part sequence of component $\Lambda_{{\pi_{k} ,h}}$ is $\delta_{{\Lambda_{{\pi_{k} ,h}} }} = \left\{ {\delta_{{\Lambda_{{\pi_{k} ,h}} ,1}} ,\delta_{{\Lambda_{{\pi_{k} ,h}} ,2}} , \ldots ,\delta_{{\Lambda_{{\pi_{k} ,h}} ,u_{{\Lambda_{{\pi_{k} ,h}} }} }} } \right\}$, where $u_{{\Lambda_{{\pi_{k} ,h}} }}$ is the number of parts belonging to component $\Lambda_{{\pi_{k} ,h}}$. An illustrative example is presented in “An illustrative example” section. The product sequence in the assembly factory is {1, 3, 2}. Three component sequences are $\Lambda_{1} = \{ 1,2\}$, $\Lambda_{2} = \{ 3,4\}$ and $\Lambda_{3} = \{ 5,6\}$. Six part sequences are $\delta_{1} = \{ 1,2\}$, $\delta_{2} = \{ 3,4\}$, $\delta_{3} = \{ 5,6\}$, $\delta_{4} = \{ 7,8\}$, $\delta_{5} = \{ 9,10\}$, $\delta_{6} = \{ 11,12\}$.

### Initial population

The initial population has an important effect on the quality of the solution for swarm intelligence algorithms. In order to improve the quality of the initial population, there are three interdependent decisions need to be dealt with for the presented DAPFSP: (1) determination of product sequence in the assembly factory; (2) allocation of components to components manufacturing factories and determination of components sequence for each product; (3) determination of part sequence for each component.

Inspired by the pioneering work of Naderi and Ruiz^11^, we assigned each component to the factory which has the minimum makespan among components manufacturing factories when including the component. To maintain the diversity of the population and save computation time, the product sequence and the part sequence for each component are generated randomly.

### Population division and weight assignation

The SSO divides the social spider population (denoted as $S$) into two different groups: female spiders (denoted as $F$) and male spiders (denoted as $M$). In the social spider population, the number of female spiders (denoted as $N_{f}$) outnumber male spiders (denoted as $N_{m}$). and $N_{f}$ usually make up 65–90% of the population size (denoted as $N$). In this paper, the female ratio is set to 70%, as in paper^45^. Therefore, $N_{f}$ is calculated according to the following formula:

17 $$N_{f} = \left| {70\% \cdot N} \right|$$

where the mathematical symbol | | can take an integer as the number of female spiders. The number of male spiders $$N_{m} = N - N_{f}$$. The population $$S = \{ s_{1} ,s_{2} , \ldots ,s_{N} \}$$ is composed of a set of female spiders ($$F = \{ f_{1} ,f_{2} , \ldots ,f_{{N_{f} }} \}$$) and a set of male individuals ($$M = \{ m_{1} ,m_{2} , \ldots ,m_{{N_{f} }} \}$$). As $$S = F \cup M$$,$$S = \{ s_{1} = f_{1} ,s_{2} = f_{2} , \ldots ,s_{{N_{f} }} = f_{{N_{f} }} ,s_{{N_{f} + 1}} = m_{1} ,$$$$s_{{N_{f} + 2}} = m_{2} ,...,s_{N} = m_{{N_{m} }} \}$$.

In the proposed three SSO algorithms, the solution quality of the individual (spider) $i$ is measured by a weight $w_{i}$. The following equation is used to calculated the weight $w_{i}$.

18-1 $$w_{i} = \frac{{worst - C_{\max } (s_{i} )}}{worst - best + c}$$

18-2 $$worst = \max (C_{\max } (s_{i} ))\quad i \in (1,2, \ldots ,N)$$

18-3 $$best = \min (C_{\max } (s_{i} ))\quad i \in (1,2, \ldots ,N)$$

18-4 $$c = 0.001$$

### Cooperative operators

The search space of the SSO is assumed as a communal web, in which social spiders update positions. In the communal web, female and male social spiders update positions according to different cooperative operators.

### Female cooperative operator

Female spiders have two behavior patterns for updating their positions: attraction movement and repulsion movement. The selection of the final behavior pattern for the female spider $f_{i}$ is influenced by three factors: the vibration $V_{c,i}$ perceived by $f_{i}$ form the nearest spider (denoted as $s_{c}$) with a better weight; the vibration $V_{b,i}$ perceived by $f_{i}$ form the best spider (denoted as $s_{b}$) of the population; and a stochastic movement. The movement of the female spider $f_{i}$ can be defined as follows:

19-1 $$f_{new,i} = \left\{ {\begin{array}{*{20}l} \begin{gathered} f_{i} + r_{1} \cdot V_{c,i} \cdot (s_{c} - f_{i} ) + r_{2} \cdot V_{b,i} \hfill \\ \quad \times (s_{b} - f_{i} ) + r_{3} (r_{4} - 0.5) \hfill \\ \end{gathered} \hfill & {r \le PF} \hfill \\ \begin{gathered} f_{i} - r_{1} \cdot V_{c,i} \cdot (s_{c} - f_{i} ) - r_{2} \cdot V_{b,i} \hfill \\ \quad \times (s_{b} - f_{i} ) + r_{3} (r_{4} - 0.5) \hfill \\ \end{gathered} \hfill & {r > PF} \hfill \\ \end{array} } \right.$$

19-2 $$V_{c,i} = w_{c} \cdot e^{{ - \sqrt {||s_{c} - f_{i} ||} }}$$

19-3 $$V_{b,i} = w_{b} \cdot e^{{ - \sqrt {||s_{b} - f_{i} ||} }}$$

where $f_{new,i}$ is the newly generated position of the female spider $f_{i}$. $r_{1}$, $r_{2}$, $r_{3}$, $r_{4}$ and $r_{5}$ are five random numbers between $\left[ {0,1} \right]$. $PF$ is a threshold which determines the selection of attraction movement or repulsion movement. In this paper, $PF$ is set to 0.7 for three proposed SSO algorithms. $w_{c}$ and $w_{b}$ are weights of the nearest spider and the best spider, respectively. The notation $|| \cdot ||$ represents the Euclidean distance. The final random item $r_{3} (r_{4} - 0.5)$ donates a stochastic movement.

### Male cooperative operator

The male spiders are divided into dominant members and non-dominant members according to weight. $m_{m}$ is the male spider whose weight is the median value of the male population. A male spider with a weight greater than the median value is assigned to dominant members; otherwise, it is regarded as one non-dominant member. Dominant male spiders tend towards the nearest female member (denoted as $f_{n}$) to generate a new generation. On the contrary, non-dominant male spiders move towards the center of the male group to utilize the remaining resources. The male cooperative operator can be formulated as follows:

$$m_{new,i} = \left\{ {\begin{array}{*{20}l} \begin{gathered} m_{i} + r_{1} \cdot V_{n,i} \cdot (f_{n} - m_{i} ) \hfill \\ \quad + r_{2} (r_{3} - 0.5) \hfill \\ \end{gathered} \hfill & {w_{i} > w_{m} } \hfill & {\left( {20{\text{-}}1} \right)} \hfill \\ {m_{i} + r_{1} \cdot (W_{mean} - m_{i} )} \hfill & {w_{i} \le w_{m} } \hfill & {\left( {20{\text{-}}2} \right)} \hfill \\ \end{array} } \right.$$

20-3 $$V_{n,i} = w_{n} \cdot e^{{ - \sqrt {||f_{n} - m_{i} ||} }}$$

20-4 $$W_{mean} = \sum\limits_{a = 1}^{{N_{m} }} {m_{a} \cdot w_{a} } /\sum\limits_{a = 1}^{{N_{m} }} {w_{a} }$$

where $m_{new,i}$ is the newly generated position of the male spider $m_{i}$. $r_{1}$, $r_{2}$ and $r_{3}$ are random numbers between $\left[ {0,1} \right]$. $V_{n,i}$ is the vibration perceived by $m_{i}$ form the nearest female spider $f_{n}$. $w_{i}$, $w_{m}$ and $w_{a}$ is the weight of $m_{i}$, $m_{m}$ and $m_{a}$, respectively. $W_{mean}$ is the weighted mean of male spiders. $w_{n}$ is the weight of the nearest female spider $f_{n}$.

The male spiders are sorted descending by their weight. The sequence after permutation is $M = \{ m_{1} ,m_{2} , \ldots ,m_{m} , \ldots ,m_{{N_{f} }} \}$. The movement of dominant male spiders and non-dominant male spiders are modeled by formulas (20-1) and (20-2), respectively.

### Mating operator

Dominant male spiders and female spiders perform mating operator in the communal web. The radius (denoted as $r$) limits the mating range of each dominant male spider. For one dominant male spider $m_{k}$, it finds out a set of female spiders (denoted as $E_{k}$) within the mating range. The mating operator is performed in set $T_{k} = E_{k} \cup m_{k}$. If $E_{k}$ is a non-empty set, the mating operator is executed to generate a new individual $m_{new}$; otherwise, the mating operator is not performed. The radius is calculated by the following formula:

21 $$r = \left\| {s_{\max } - s_{\min } } \right\| \cdot ratio$$

where $s_{\max }$ and $s_{\min }$ represent the boundary of search space. Recall the presented solution representation, there are $s_{\max } = \{ p,p - 1,p - 2, \ldots ,1\}$ and $s_{\min } = \{ 1,2, \ldots ,p\}$. $ratio \in (0,1)$ is a factor controlling the radius $r$.

If the new individual $m_{new}$ is generated, compare its weight with the weight of the worst individual in the population. If the new individual $m_{new}$ is better than the worst individual, the worst individual is replaced by $m_{new}$, and $m_{new}$ inherits the gender and index of the replaced individual. Otherwise, the new individual $m_{new}$ is ignored and the population remain unchanged.

### Neighborhood operators and local search strategies

Neighborhood operators play an important role in designing a heuristic algorithm and it can enhance the exploitation ability of the algorithm. According to the representation, there are three types of neighborhoods. The first one is based on the product sequence, the second type is based on the component sequence, the third kind is based on the part sequence. For one solution, we can shift its product sequence and keep the other two types of sequences unchanged. Shift operators are widely used in scheduling problems. A shift operator moves one element in a sequence to another location and keeps the other elements in the same position. Considering a product sequence $\pi = \left\{ {\pi_{1} ,\pi_{2} , \ldots ,\pi_{k} ,\pi_{k + 1} , \ldots ,\pi_{\alpha } } \right\}$, the first product $\pi_{1}$ can be shifted to the $k^{th}$ position and generating a new sequence $\pi_{new} = \left\{ {\pi_{2} , \ldots ,\pi_{k} ,\pi_{1} ,\pi_{k + 1} , \ldots ,\pi_{\alpha } } \right\}$, while keeping the component sequence of each product and part sequence of each component unchanged. The same shift operator applies to the component-based neighborhood and part-based neighborhood.

According to the presented three neighborhood operators, we design three local search strategies. The first local search strategy is designed based on the neighborhood operator of the produce sequence. For an individual $s_{i}$ with a product sequence $\pi = \left\{ {\pi_{1} ,\pi_{2} , \ldots ,\pi_{k} ,\pi_{k + 1} , \ldots ,\pi_{\alpha } } \right\}$, the product $\pi_{k}$ can be inserted into $\alpha - 1$ possible positions and generate $\alpha - 1$ different neighborhood solutions. $s_{i}^{*}$ is the best solution of all the $\alpha - 1$ neighborhood solutions. If $s_{i}^{*}$ is better than $s_{i}$, replace $s_{i}$ with $s_{i}^{*}$. Otherwise, $s_{i}$ remains unchanged. The above process is performed for each product in the product sequence. The procedure of product-based local search strategy is presented in Algorithm 1.

**Figure:**
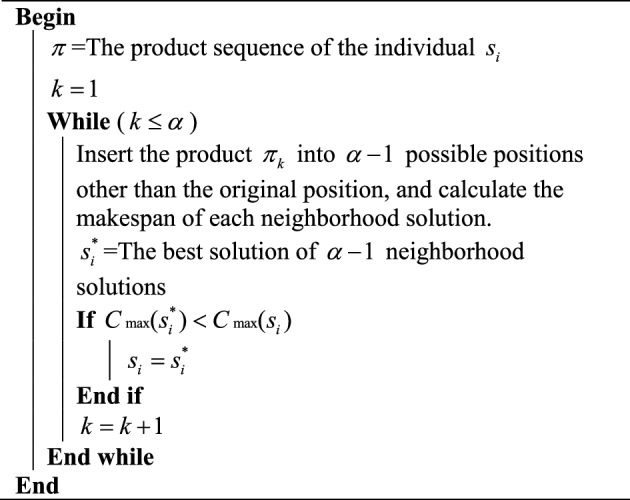
Algorithm 1 Product-based local search

The second local search strategy is designed based on component sequences. $\Lambda_{{\pi_{k} }} = \{ \Lambda_{{\pi_{k} ,1}} ,\Lambda_{{\pi_{k} ,2}} , \ldots ,\Lambda_{{\pi_{k} ,h}} ,\Lambda_{{\pi_{k} ,h + 1}} , \ldots ,\Lambda_{{\pi_{k} ,n_{{\pi_{k} }} }} \}$ is the component sequence of product $\pi_{k}$, where $n_{{\pi_{k} }}$ is the number of components belonging to product $\pi_{k}$. The component $\Lambda_{{\pi_{k} ,h}}$ can be inserted into $n_{{\pi_{k} }} - 1$ possible positions and produce $n_{{\pi_{k} }} - 1$ different neighborhood solutions. $s_{i}^{*}$ is the best solution of all the $n_{{\pi_{k} }} - 1$ neighborhood solutions. If $s_{i}^{*}$ is better than $s_{i}$, replace $s_{i}$ with $s_{i}^{*}$. Otherwise, $s_{i}$ remains unchanged. The above process is performed for each component of the product $\pi_{k}$. The procedure of component-based local search strategy is illustrated in Algorithm 2.

**Figure:**
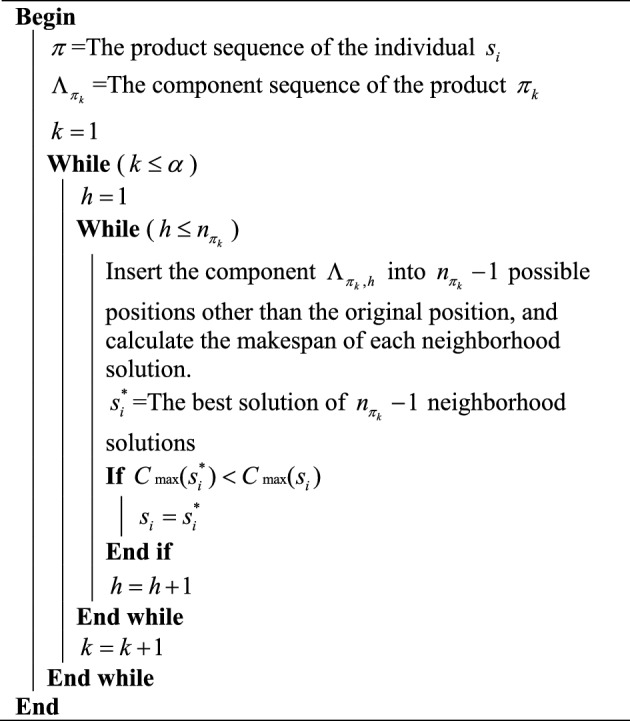
Algorithm 2 Component-based local search

The third local search strategy is designed based on part sequences. The part sequence of component $\Lambda_{{\pi_{k} ,h}}$ is $\delta_{{\Lambda_{{\pi_{k} ,h}} }} = \left\{ {\delta_{{\Lambda_{{\pi_{k} ,h}} ,1}} ,\delta_{{\Lambda_{{\pi_{k} ,h}} ,2}} ,...,\delta_{{\Lambda_{{\pi_{k} ,h}} ,j}} ,\delta_{{\Lambda_{{\pi_{k} ,h}} ,j + 1}} ,...,\delta_{{\Lambda_{{\pi_{k} ,h}} ,u_{{\Lambda_{{\pi_{k} ,h}} }} }} } \right\}$, where $u_{{\Lambda_{{\pi_{k} ,h}} }}$ is the number of parts belonging to component $\Lambda_{{\pi_{k} ,h}}$. The part $\delta_{{\Lambda_{{\pi_{k} ,h}} ,j}}$ can be inserted into $u_{{\Lambda_{{\pi_{k} ,h}} }} - 1$ possible positions and can produce $u_{{\Lambda_{{\pi_{k} ,h}} }} - 1$ different neighborhood solutions. $s_{i}^{*}$ is the best solution of all the $u_{{\Lambda_{{\pi_{k} ,h}} }} - 1$ neighborhood solutions. If $s_{i}^{*}$ is better than $s_{i}$, replace $s_{i}$ with $s_{i}^{*}$. Otherwise, $s_{i}$ remains unchanged. The above process is performed for each part of the component $\Lambda_{{\pi_{k} ,h}}$. The procedure of part-based local search strategy is shown in Algorithm 3.

**Figure:**
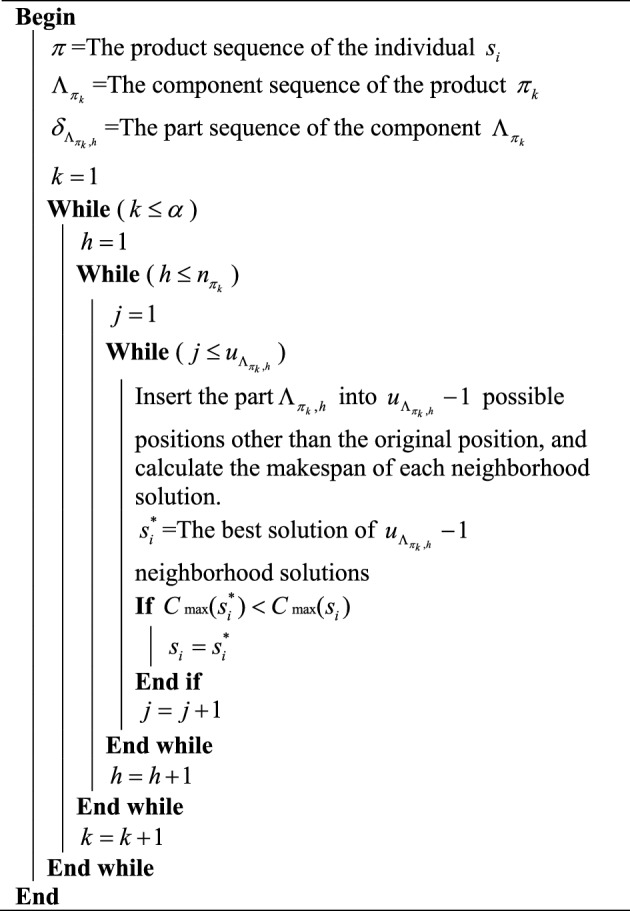
Algorithm 3 Part-based local search

### Restart procedure

#### Opposition-based restart procedure

In order to maintain the diversity of the population and avoid premature convergence, we introduce an opposition-based restart (OBR) procedure that combines restart procedure with opposition-based learning (OBL). The OBL was proposed by Tizhoosh^50^ and its effective has been proved in optimization problems^51–55^. Xu et al.^56^ described in detail the concept of OBL, and the process of generating opposite numbers is presented as follows.

##### Definition 1

Let $x \in [a,b]$ be a real number. The opposite number $\tilde{x}$ can be defined by

22 $$\tilde{x} = a + b - x.$$

Similarly, the opposite vector for a D-dimensional vector can be defined as follows:

##### Definition 2

Let $X = (x_{1} ,x_{2} , \ldots ,x_{D} )$ be a D-dimensional vector, where $x_{i} \in [a_{i} ,b_{i} ]$, $i \in 1,2,...,D$. the opposite vector $\tilde{X} = (\tilde{x}_{1} ,\tilde{x}_{2} , \ldots ,\tilde{x}_{D} )$ can be defined by

23 $$\tilde{x}_{i} = a_{i} + b_{i} - x_{i} .$$

For example, there is the product permutation $\pi = \{ 3,2,7,6,8,1,9,4,5\}$ consists of 9 products. According to the definition 2, the opposite product permutation can be calculated as $\tilde{\pi } = \{ 7,8,3,4,2,9,1,6,5\}$.

The minimum makespan of each iteration is stored. If the minimum is not improved in ten consecutive iterations, we adopt the following OBR procedure to increase population diversity.

*Step 1* Sort all spiders in the population in descending order of weight.

*Step 2*: Take the top 10% of individuals and calculate their opposite positions based on the sequence of products.

*Step 3* An opposite position is accepted if the makespan is better, while a poor opposite position (denoted as $\tilde{\pi }$) is accepted according to the following probability $p_{{\tilde{\pi }}}$:

24 $$p_{{\tilde{\pi }}} = \frac{{worst - C_{\max } (\tilde{\pi })}}{worst - best + c}.$$

*Step 4* Among the remaining 90% individuals, randomly select the same number of individuals as the opposite positions, and replace the selected individuals with the opposite positions.

#### Inverse-based restart procedure

**Figure 3:**
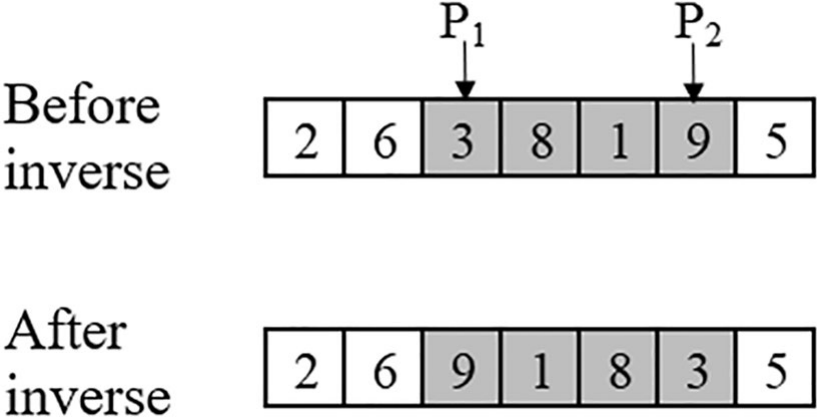
An example of the inverse operator.

Since the permutation of components and parts is not a set of consecutive integers, the OBL cannot be used directly. For the component-based sequence and part-*based* sequence, we apply an inverse operation to generate new permutations. The procedure of inverse operator is shown as follows: (a) randomly select two points *P*_1_ and *P*_2_ of the permutation; (b) reverse the sequence between *P*_1_ and *P*_2_. Figure 3 presents an instance of the inverse operator. The procedure of inverse-based restart (IBR) is shown as follows:

*Step 1* Sort all spiders in the population in descending order of weight.

*Step 2* Take the top 10% of individuals and calculate their inverse neighborhoods based on the sequence of components and parts.

*Step 3* An inverse position is accepted if the makespan is better, while a poor inverse position (denoted as $\tilde{\pi }$) is accepted according to the formula (24).

*Step 4* Among the remaining 90% individuals, randomly select the same number of individuals as the inverse neighborhoods, and replace the selected individuals with the opposite positions.

### SSO with hybrid local search strategies

Three local search strategies mentioned above are quite different, and they can enhance the search ability of discrete SSO. In order to make full use of the advantages of these strategies and obtain the optimal solution or the near optimal solution, we propose a hybrid SSO (HSSO for short) that adopts these strategies simultaneously. The procedure of HSSO is illustrated in Algorithm 4.

**Figure:**
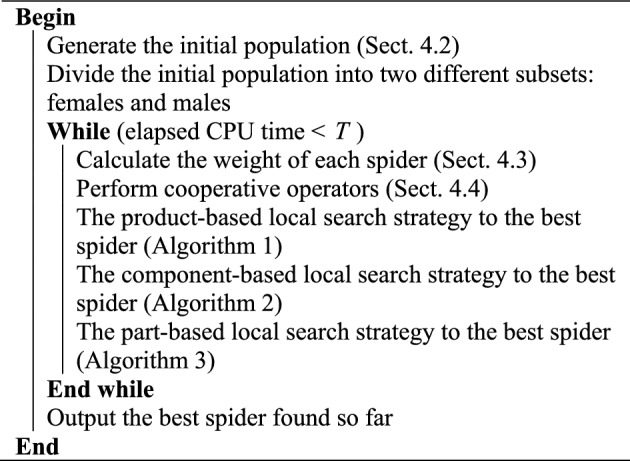
Algorithm 4 HSSO

### HSSO with restart procedures

With the evolution of HSSO, an increasing number of individuals have a tendency to converge together, so the algorithm may be trapped in local optimal. To maintain the diversity of the population and avoid premature convergence, we present the HSSO with two restart procedures (HSSOR for short). The procedure of HSSOR is shown in Algorithm 5.

**Figure:**
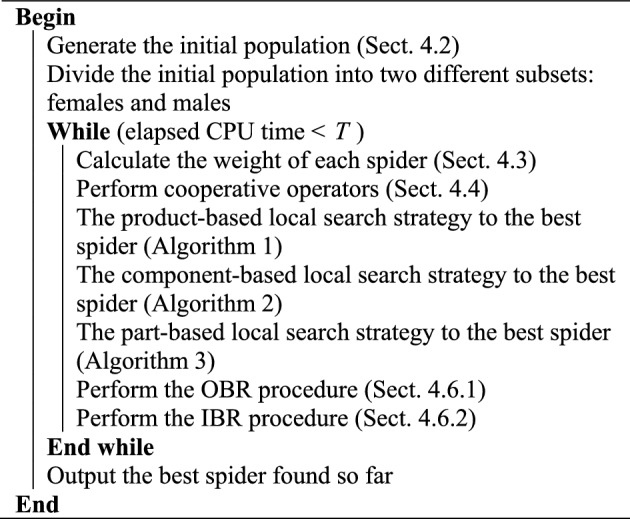
Algorithm 5 HSSOR

### HSSOR with self-adaptive selection probability

Since the OBR and product-based local search strategy operate the product sequence, and a product is composed of a series of components and parts, they are beneficial to enhance the exploration ability of the algorithm. The IBR and component-based local search strategy and the part-based local search strategy are based on shifting of components and parts, so they can improve the exploitation ability of the algorithm. The OBR and product-based local search strategy are more needed in the early generations, because almost all individuals are far from the optimal solution. In the final iterations, some individuals of the population are close to the optimum solution or near optimal solutions. The IBR and component-based and part-based local search strategies are more needed. To balance exploration and exploitation, a self-adaptive selection probability (denoted as $P_{sa}$) is introduced to control the use of two restart procedures and three local search strategies. The parameter $P_{sa}$ is defined by the following formula:

25 $$P_{sa} = \rho_{\min } + \frac{t}{T} \cdot (\rho_{\max } - \rho_{\min } )$$

where $t$ and $T$ are the current elapsed time and maximum elapsed time respectively. $\rho_{\min }$ and $\rho_{\max }$ are decimals between 0 and 1. The component-based and part-based local strategies are adopted with probability $P_{sa}$. Otherwise, the product-based local search strategy is used. Algorithm 6 illustrates the procedure of HSSO with self-adaptive selection probability (HSSORP for short).

**Figure:**
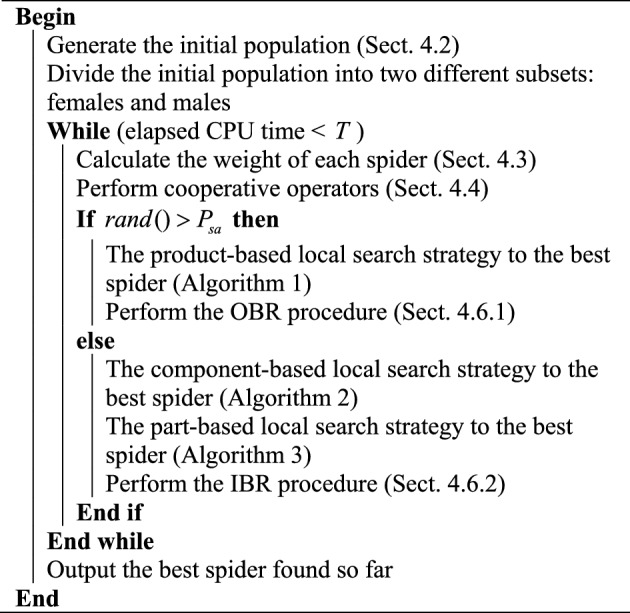
Algorithm 6 HSSORP

## Experiment analyses

### Experimental design

Since the DAPFSP presented in this paper is an extension of the innovative work of Hatami et al.^12,13^ and Pan et al.^24^, the instances of the proposed problem are extended based on the 810 large instances of Hatami et al.^12^. We set instances with the number of parts {100, 200, 500}, the number of machines in the production stage $M \in${5, 10, 20}, the number of components manufacturing factories $F \in${4, 6, 8}, the number of components $\beta \in${30, 40, 50}. To satisfy the assumption that each product is composed of at least two components and limit the number of instances, we assign 30 components, 40 components and 50 components to 10 products, 15 products and 20 products, respectively. For each instance, all time indexes are set to integers. The processing time of each part in different product stages is fixed to *U*[1, 99]. The assembly time of each component is set to *U*[$1 \times n$, $99 \times n$], where $n$ is the number of parts belonging to the component.

The assembly time of each product in the assembly factory is set to *U*[$1 \times n$, $99 \times n$], where $n$ is the number of components belonging to the product. There are $3 \times 3 \times 3 \times 3$ = 81 different combinations and each combination has 10 replications. The 810 instances zip file is available. For example, the notation “I_100_5 _4_30_10_1” means an instance consists of 100 parts, 5 machines, 4 components manufacturing factories, 30 components and 10 products. The last number ‘1’ of the notation indicates that the instance is the first one of the combination.

The relative percentage deviation (*RPD*) is used to measure solutions obtained by different algorithms. The formula for calculating *RPD* is as follows:

26 $$RPD = \frac{{C_{\max } - C_{best} }}{{C_{best} }} \times 100$$

where is $C_{\max }$ the makespan calculated by one algorithm, and $C_{best}$ is the minimum makespan obtained by all comparing algorithms.

### Experimental calibration

In this subsection, we calibrate the proposed three SSO variants and comparing algorithms. This study is the first work to solve the DAPFSP with the assumption that each component of the final product is composed of several parts. This kind of problem is widespread in the actual production process, especially in auto parts supply chain, but there is no published works to make comparisons. To verify the validity of the proposed three algorithms, we conduct a comparison with two excellent work on the distributed flowshop scheduling problem including CMA^17^ and EDA^19^. By incorporating the presented solution representation and the above-mentioned objective function, we adopt CMA and EDA to the proposed DAPFSP.

The parameters of the algorithm affect its performance. To calibrate these five algorithms, we generate an instance for each combination randomly and employ Taguchi method^57^. To set parameters effectively, we conducted preliminary experiments. For the HSSORP, the population size $P_{s}$, the levels of the self-adaptive selection probability $P_{sa}$ ($\rho_{\min }$–$\rho_{\max }$), and the parameter $ratio$ of mating operator, are shown in Table 2.

**Table 2:** Parameter values.

Parameter	Values			
	1	2	3	4
*P*_s_	50	100	150	200
$P_{sa}$($\rho_{\min }$–$\rho_{\max }$)	0.1–0.5	0.2–0.8	0.2–0.9	0.3–0.8
Ratio	0.6	0.7	0.8	0.9

**Figure 4:**
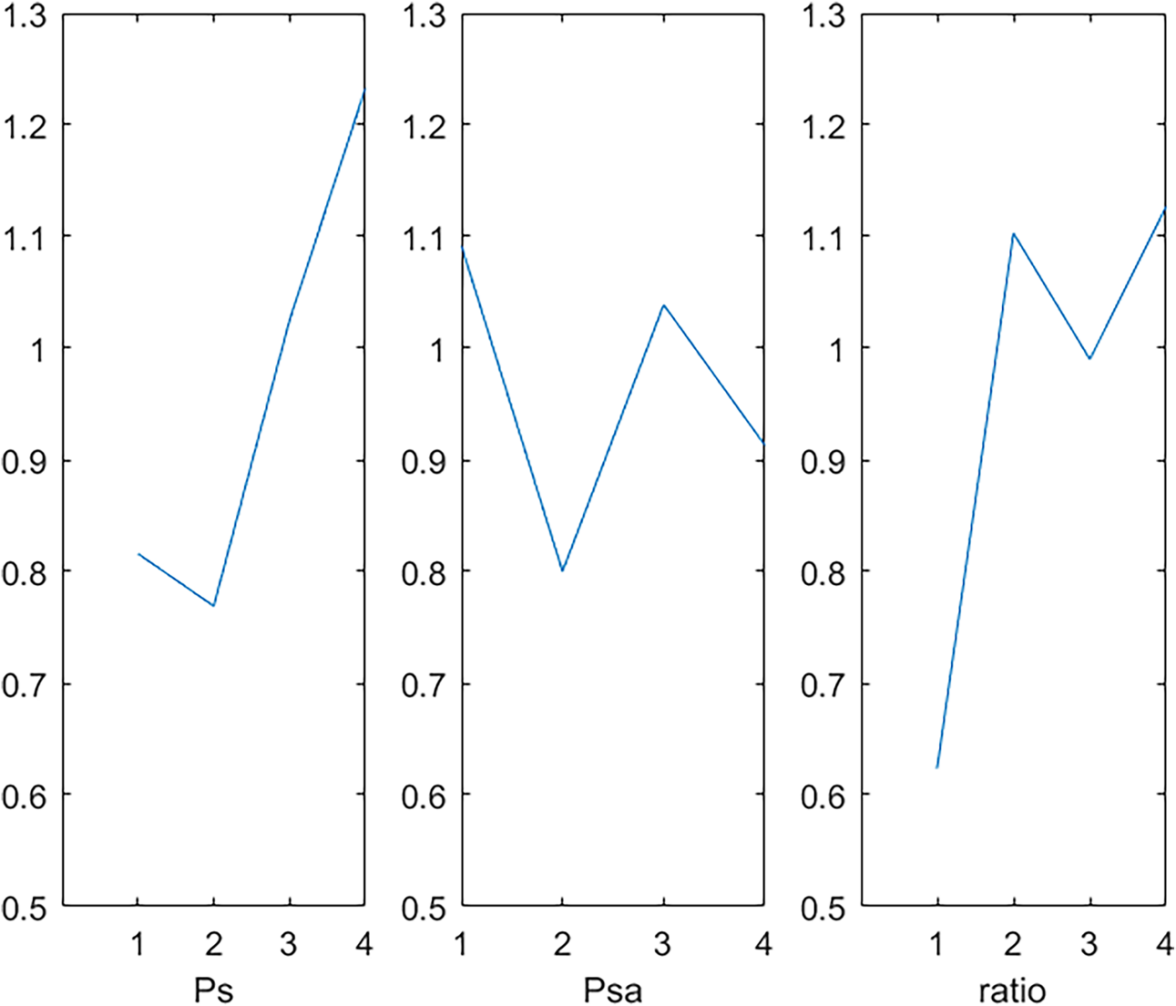
Factor level trend.

We use C++ o code the HSSOPR in VS 2015 and the elapsed CPU time is set to ${20} \times \chi \times M$ milliseconds. For three critical parameters, we chose the orthogonal array L_16_ (4^3^) that test 16 combinations of different parameter levels. The HSSOPR runs 20 times independently for each combination on a computer with 8 Intel(R) Core (TM) i5-10210U CPU @ 1.60GHz, 8 GB RAM and Windows 10 operation system. The average *RPD* (*aRPD*) values is calculated and presented in Table 3. The parameter level trend is shown in Fig. 4. According to Fig. 4, HSSORP performs better with the following parameters: *P*_s_ = 100, $\rho_{\min }$ = 0.2, $\rho_{\max }$ = 0.8, *ratio* = 0.6. The other four algorithms are calibrated by the same method, and Table 4 details parameters of all five algorithms.

**Table 3:** Response values.

*P*_s_	*P*_*c*_	*C*_n_	*aRPD*
1	1	1	0.564
1	2	2	0.833
1	3	3	0.922
1	4	4	0.945
2	1	2	0.856
2	2	1	0.467
2	3	4	1.020
2	4	3	0.733
3	1	3	1.327
3	2	4	0.923
3	3	1	0.671
3	4	2	1.180
4	1	4	1.620
4	2	3	0.978
4	3	2	1.542
4	4	1	0.792

**Table 4:** Parameters of five algorithms.

Algorithms	Parameter values
HSSO	*P*_s_ = 100, *ratio* = 0.6
HSSOR	*P*_s_ = 100, *ratio* = 0.6
HSSORP	*P*_s_ = 100, $\rho_{\min }$ = 0.2, $\rho_{\max }$ = 0.8, *ratio* = 0.6
CMA	*P*_s_ = 50, *LS* = 100
EDA	*P*_s_ = 50, *γ* = 0.2

### Computational evaluation

We code all the algorithms using C++ in VS 2015 and solve the 810 instances on the above-mentioned computer. To make the experiment fair, instead of using the number of iterations as the termination condition, we let each algorithm run independently for the same time. Termination is triggered when the algorithm reaches the maximum elapsed CPU time *t* = $\tau \times \chi \times M$ milliseconds and the parameter $\tau$ is set to is set to three values 20, 40, and 60 respectively. In the given three different termination times, each algorithm runs 20 times independently for 810 instances. The best solutions of 810 instances calculated by each algorithm can be accessed. Tables 5, 6 and 7 presents the summarized *aRPD* values of three terminations.

**Table 5:** *aRPD* at CPU time *t* = ${20} \times \chi \times M$ ms.

	HSSO	HSSOR	HSSORP	CMA	EDA
Parts ($\chi$)					
100	0.628	**0.454**	0.459	1.542	6.209
200	0.106	**0.064**	0.083	2.805	13.877
500	0.094	**0.050**	0.052	3.400	16.659
Components ($\beta$)					
30	0.246	**0.156**	0.187	2.923	11.754
40	0.297	0.235	**0.201**	2.685	12.691
50	0.286	**0.177**	0.207	2.139	12.300
Machines ($M$)					
5	0.352	**0.272**	0.285	2.672	12.403
10	0.300	**0.178**	0.183	2.567	12.181
20	0.177	**0.119**	0.127	2.509	12.160
Factories ($F$)					
4	0.226	0.152	**0.150**	2.622	12.369
6	0.249	0.164	**0.157**	2.541	12.258
8	0.354	**0.251**	0.287	2.584	12.118
Products ($\alpha$)					
10	0.246	**0.156**	0.187	2.923	11.754
15	0.297	0.235	**0.201**	2.685	12.691
20	0.286	**0.177**	0.207	2.139	12.300
Means	0.276	**0.189**	0.198	2.582	12.248

Better results are in bold.

**Table 6:** *aRPD* at CPU time *t* = ${40} \times \chi \times M$ ms.

	HSSO	HSSOR	HSSORP	CMA	EDA
Parts ($\chi$)					
100	0.469	**0.318**	0.336	1.588	5.861
200	0.118	**0.076**	**0.076**	2.581	13.175
500	0.117	0.088	**0.072**	3.174	16.071
Components ($\beta$)					
30	0.197	0.144	**0.143**	2.672	11.358
40	0.258	**0.162**	0.190	2.604	12.114
50	0.248	0.176	**0.151**	2.067	11.636
Machines ($M$)					
5	0.266	**0.223**	0.241	2.499	11.719
10	0.235	**0.134**	0.138	2.486	11.647
20	0.203	0.126	**0.104**	2.358	11.742
Factories ($F$)					
4	0.181	0.137	**0.132**	2.487	11.843
6	0.221	**0.143**	0.160	2.393	11.720
8	0.302	0.202	**0.192**	2.463	11.544
Products ($\alpha$)					
10	0.197	0.144	**0.143**	2.672	11.358
15	0.258	**0.162**	0.190	2.604	12.114
20	0.248	0.176	**0.151**	2.067	11.636
Means	0.235	**0.161**	**0.161**	2.448	11.703

Better results are in bold.

**Table 7:** *aRPD* at CPU time *t* = ${60} \times \chi \times M$ milliseconds.

	HSSO	HSSOR	HSSORP	CMA	EDA
Parts ($\chi$)					
100	0.239	**0.111**	0.133	2.108	5.561
200	0.186	**0.134**	**0.134**	2.131	12.397
500	0.256	0.130	**0.185**	2.683	15.182
Components ($\beta$)					
30	0.242	**0.113**	0.131	2.391	10.874
40	0.209	**0.137**	0.184	2.423	11.452
50	0.231	**0.124**	0.137	2.107	10.814
Machines ($M$)					
5	0.235	**0.128**	0.165	2.489	11.122
10	0.222	**0.125**	0.150	2.295	10.959
20	0.224	**0.122**	0.137	2.136	11.059
Factories ($F$)					
4	0.223	**0.119**	0.139	2.227	11.430
6	0.214	**0.148**	0.155	2.305	11.044
8	0.245	**0.107**	0.158	2.389	10.666
Products ($\alpha$)					
10	0.242	**0.113**	0.131	2.391	10.874
15	0.209	**0.137**	0.184	2.423	11.452
20	0.231	**0.124**	0.137	2.107	10.814
Means	0.227	**0.125**	0.151	2.307	11.047

Better results are in bold.

Table 5 reports the *aRPD* values with CPU time *t* = ${20} \times \chi \times M$ milliseconds. It can be seen from the last row that HSSOR and HSSORP performs better than HSSO, which proves the effectiveness of the presented restart procedures. There is only a small difference in the performance of HSSOR and HSSORP. EDA gets the worst performance with 12.248% *aRPD*, which is almost 65 times that of HSSOR. This is because the search method of EDA may not be suitable for the proposed problem. CMA performs much better than EDA. Moreover, the proposed three algorithms outperform the comparison algorithms. Tables 6 and 7 present the *aRPD* values with CPU time *t* = ${40} \times \chi \times M$ milliseconds and CPU time *t* = ${60} \times \chi \times M$ milliseconds respectively. As we can see from two tables, as running time increases, our proposed algorithm can maintain a very large lead in performance. At CPU time *t* = ${40} \times \chi \times M$ milliseconds, HSSOR and HSSORP are almost equal, while HSSORP outperforms HSSOR at CPU time *t* = ${60} \times \chi \times M$ milliseconds.

**Figure 5:**
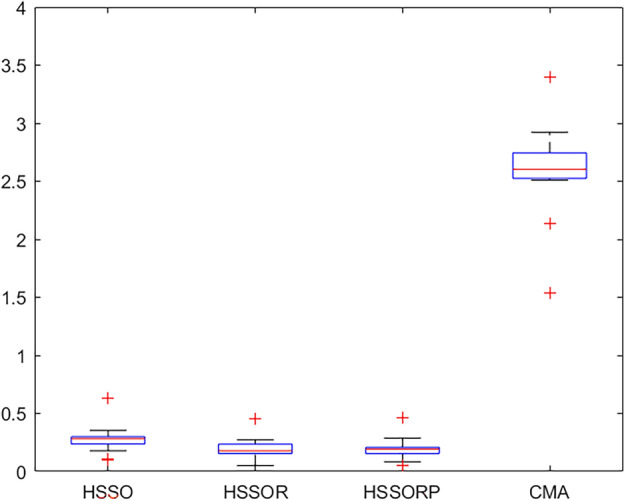
Box plots for four comparison algorithms at CPU time t = ${20} \times \chi \times M$ ms.

**Figure 6:**
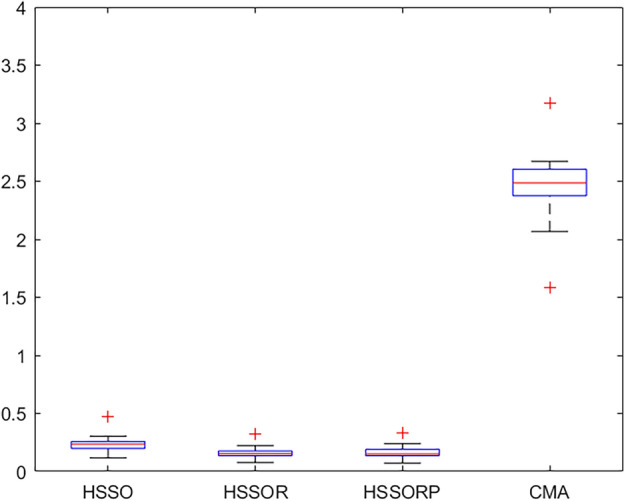
Box plots for four comparison algorithms at CPU time t = ${40} \times \chi \times M$ ms.

**Figure 7:**
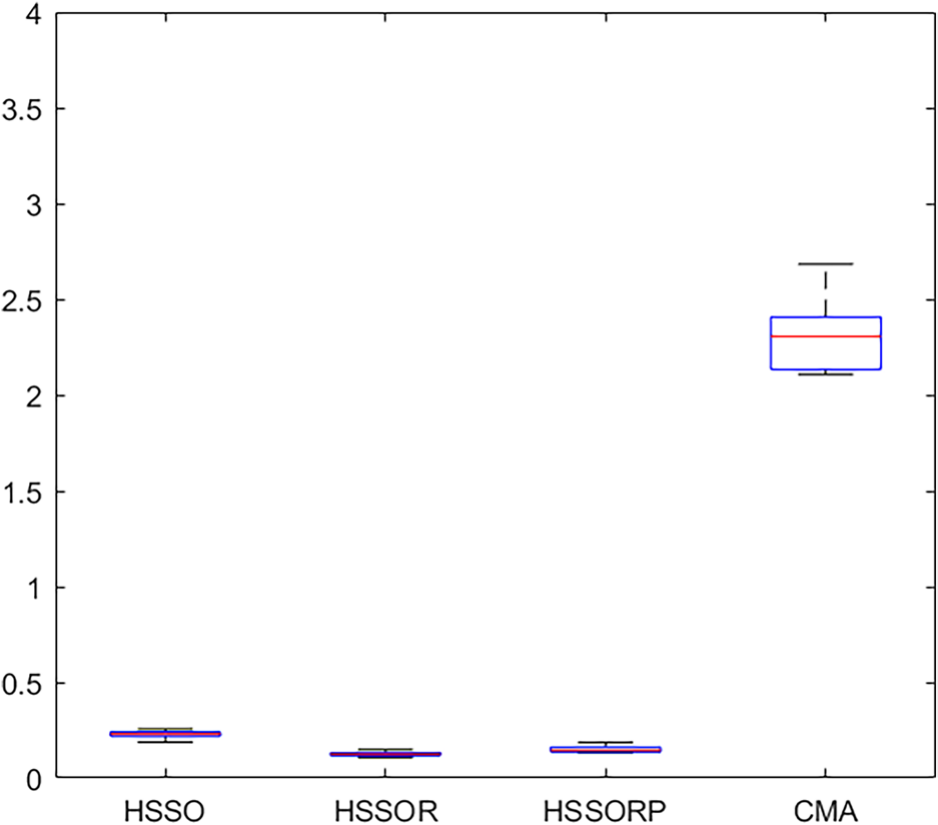
Box plots for four comparison algorithms at CPU time t = ${60} \times \chi \times M$ ms.

We analyze the results closely. EDA is taken out of the analysis because it is not suitable for the proposed problem. Figures 5, 6 and 7 present box plots for four comparison algorithms at three diffident CPU times. It can be clearly observed that: (1) statistically, our algorithms are much better than CMA, and this can prove that our strategies and algorithms is effective; (2) HSSOR and HSSORP is better than HSSO, and this can illustrate the effectiveness of the proposed restart procedures; (3) HSSOR is slightly better than HSSORP, and this can show that using three local search strategies simultaneously can achieve better results.

**Figure 8:**
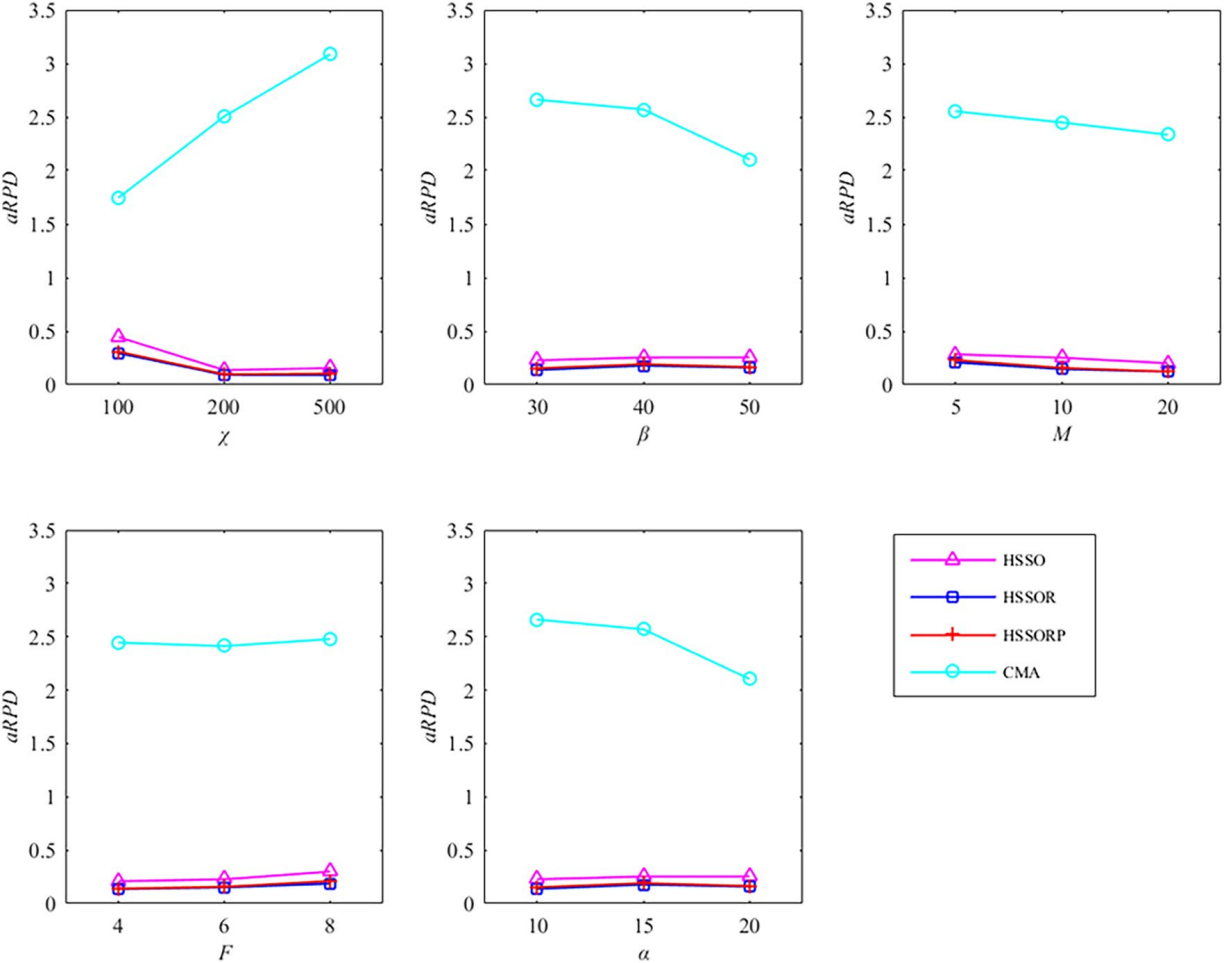
Variation trend of *aRPD* for different value levels of five parameters.

In addition, five parameters including the number of parts *χ*, the number of components *β*, the number of machines *M*, the number of factories *F*, and the number of products *α* determine the proposed problem. We conduct data analysis to figure out the effects of these parameters on the four comparison algorithms. Figure 8 shows variation trend of *aRPD* for different value levels of five parameters. It can be seen from Fig. 8 that both of HSSOR and HSSORP have very slight fluctuations and small values which illustrate that both of them have excellent robustness and performance. According to the data, all values of HSSOR for each parameter are smaller than HSSORP, which indicates HSSOR is better than HSSORP.

**Figure 9:**
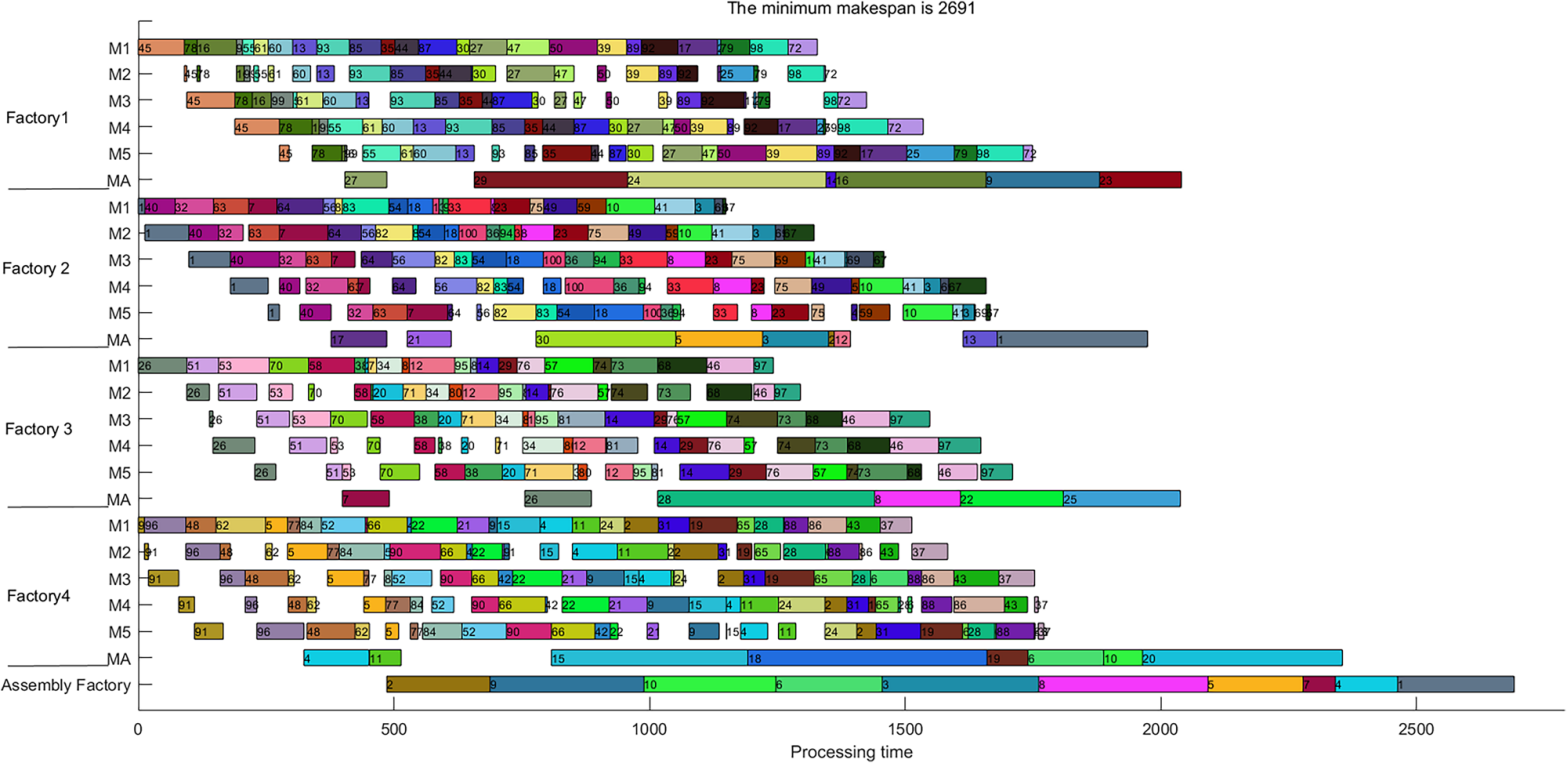
Gantt chart of instance “I_100_5_4_30_10_1”.

Finally, to illustrate the proposed DAPFSP intuitively, Fig. 9 reports the Gantt chart of instance “I_100_5_4_30_10_1” with the best solution we found so far.

## Conclusions

In conclusion, our study makes several contributions from different perspectives: theoretical, managerial, and practical. (i) Theoretical contribution: This paper presents the first study on DAPFSP considering the intricate nature of modern supply chains, where components of one final product are manufactured by multiple companies. Our proposed problem formulation requires determining the optimal product sequence in the assembly factory, as well as the component sequence and part sequence in multiple component manufacturing factories. To address this challenge, we introduce a novel three-level representation and an initialization method, along with three local search methods to enhance the algorithm's search ability. Additionally, to prevent premature convergence, we design two restart procedures tailored to the problem's characteristics. Furthermore, we propose three discrete SSO algorithms for the proposed DAPFSP. Our experiments, calibrated using the Taguchi method, demonstrate the efficiency of these algorithms in solving the problem. (ii) Our study provides insights into the critical role of efficient scheduling in supply chain management. By addressing a crucial aspect of production processes, it partially bridges the gap between academic research and practical applications. However, it is important to acknowledge that our research does not encompass all constraints present in practical production scenarios, such as transportation costs^58^, production capacity constraints, setup times, machine maintenance, and rescheduling in emergencies^59^. These aspects are vital considerations for real-world applications and should be incorporated into future research endeavors. (iii) In the view of practical standpoint, our research sets the stage for further exploration into the integration of manufacturing processes in Industry 4.0^60^. Moving forward, we are committed to addressing the aforementioned constraints and designing efficient scheduling algorithms that can significantly enhance production efficiency in practical settings. By focusing on the challenges encountered in real-world production environments, we aim to contribute to the advancement of manufacturing processes and facilitate the transition towards Industry 4.0.

## Data Availability

The datasets used and/or analyzed during the current study available from the corresponding author on reasonable request.
